# Infections in DNA Repair Defects

**DOI:** 10.3390/pathogens12030440

**Published:** 2023-03-10

**Authors:** Yesim Yilmaz Demirdag, Sudhir Gupta

**Affiliations:** Division of Basic and Clinical Immunology, Department of Medicine, University of California, Irvine, CA 92617, USA

**Keywords:** inborn errors of immunity, immunodeficiency, DNA repair disorders, ataxia telangiectasia, infections, Nijmegen breakage syndrome, Bloom syndrome

## Abstract

DNA repair defects are heterogenous conditions characterized by a wide spectrum of clinical phenotypes. The common presentations of DNA repair defects include increased risk of cancer, accelerated aging, and defects in the development of various organs and systems. The immune system can be affected in a subset of these disorders leading to susceptibility to infections and autoimmunity. Infections in DNA repair defects may occur due to primary defects in T, B, or NK cells and other factors such as anatomic defects, neurologic disorders, or during chemotherapy. Consequently, the characteristics of the infections may vary from mild upper respiratory tract infections to severe, opportunistic, and even fatal infections with bacteria, viruses, or fungi. Here, infections in 15 rare and sporadic DNA repair defects that are associated with immunodeficiencies are discussed. Because of the rarity of some of these conditions, limited information is available regarding infectious complications.

## 1. Introduction

DNA damage can occur spontaneously or because of environmental exposure to various agents such as ultraviolet radiation, ionizing radiation, and chemicals, including alkylating agents, aromatic amines, and cross-linking agents [1]. Cells utilize several DNA repair mechanisms to avoid deleterious consequences of DNA damage, which may result in mutations and genomic instability. These mechanisms include DNA mismatch repair (MMR), base excision repair (BER), nucleotide excision repair (NER), single-strand break repair (SSBR), and double-strand break repair (DSBR) [2]. In general, DNA repair starts with damage recognition, activation of checkpoint proteins, and finally, activation of repair enzymes such as nucleases, helicases, polymerases, and ligases. Double-strand breaks are the most toxic DNA breaks resulting in cell death or large-scale gene rearrangement, causing cancer if not properly repaired. The two main DSBR pathways are homologous recombination (HR) and nonhomologous end-joining (NHEJ) pathways. HR is the most accurate because it uses the homologous sister chromatid as a template. Disorders of DNA repair mechanisms may result from alterations in these repair pathways, causing genomic instability and various disease phenotypes ranging from neurodevelopmental syndromes, immunodeficienciencies, increased risk of a variety of malignancies, to premature aging [3].

In addition to a spontaneous occurrence or triggered by environmental factors, in some cells, DNA breaks are programmable and necessary. A classic example of programmed DNA breaks occurs during somatic rearrangement of T cell receptors (TCR) and immunoglobulin receptor (or B cell receptors-BCR) genes, a process called V(D)J recombination, which is essential in the generation of diverse antigenic repertoire. DNA repair mechanisms play a crucial role in the development of these antigen receptors, which are critical for normal immune response. Defects in DNA repair pathways may lead to various alterations in the development and maturation of T and B cells, causing susceptibility to recurrent or severe infections [4]. The majority of DNA repair defects that are most associated with immunodeficiencies are due to defects in 3 repair pathways: DSBR, MMR (such as LIG4 deficiency, ataxia telangiectasia, and Nijmegen Breakage syndrome) and BER (UNG deficiency). DNA repair defects associated with notable immunodeficiencies are included in the classification of inborn errors of immunity (IEI) under several categories, mainly severe combined immunodeficiencies, combined immunodeficiencies, and bone marrow failure syndromes [5].

In this review, 15 IEIs associated with DNA repair defects and their infectious manifestations are discussed and summarized in Table 1. Disorders that are listed in the most recent classification by the international union of immunological societies (IUIS) are reviewed [5]. Other DNA repairs defects, such as DNA ligase IV deficiency, Artemis deficiency, and Cernunnose/XLF deficiency, are discussed in chapter 1 (Infections in SCID). Two extremely rare defects, PMS2 deficiency, and Hebo deficiency, are included in the table only because infections have not been reported or characterized in these conditions [6,7].

## 2. Ataxia-Telangiectasia

Ataxia Telangiectasia (AT) is a rare autosomal recessive (AR) disorder affecting multiple systems, primarily the nervous system, and immune system, with a median life expectancy of 25 years [8]. It is caused by mutations in the *ATM* (ataxia–telangiectasia, mutated) gene that encodes the protein ATM, a protein kinase that plays a critical role in DSBR. 

In addition to the result of exposure to ionizing radiation and other external factors, double-strand breaks (DSB) occur during TCR as well as BCR gene rearrangements. When ATM does not function properly, the cell cycle does not stop to repair DSB. This results in defects in T cell receptor (TCR) and B cell receptor (BCR) rearrangement, which ultimately causes defects in the development of T and B cells [9,10]. Prevalence, severity, and type of immunologic abnormalities in AT are highly variable and usually associated with lymphopenia, low immunoglobulin levels (IgG/IgA or IgM), low IgG2 or IgG3 and suboptimal polysaccharide antibody responses. Despite low CD4+ T cell counts, T cell function is usually preserved [10,11,12]. About 10–20% of patients with AT present with hyper-IgM phenotype, which is likely due to a blockade in early B cell development resulting in immunoglobulin class-switching defect (CSD) [13,14]. Importantly, the hyper-IgM phenotype is associated with more severe infectious manifestations and lower survival rates [13,15].

The symptoms of AT usually develop early in the first 1–2 years of life and include progressive cerebellar ataxia, which is the most reported presentation [16]. Other neurologic symptoms include the progressive development of dysarthria, dysphagia, oculomotor apraxia, dystonia, tremor, and peripheral neuropathy. Some patients may also have progressive cognitive impairment. By 10 years of age, the majority of children are unable to walk. Telangiectasia often involves the bulbar conjunctivae, pinna of the ears, and other places in the body starting around 3–4 years of age. Frequent infections may start as early as the first months of life and mostly involves infections of the upper and lower respiratory tracts requiring prophylactic antibiotic therapy as well as immunoglobulin replacement therapy [10,11]. In contrast to progressive neurologic disease, progressive immunodeficiency is very rare in AT [10,17]. In addition to neurologic deterioration and infections, increased risk of malignancies, particularly leukemia, and lymphoma, and increased risk of toxicity associated with chemo- and radiation therapies further impact life expectancy in AT [16,18].

### 2.1. Respiratory Tract Infections in AT

In a large cohort of patients with AT, recurrent upper respiratory tract infections were reported by more than one-third of patients. Due to progressive neurologic disease, which results in respiratory failure, the prevalence of lower respiratory tract infections increases with age and may be seen in up to 38% of patients older than 20 years [10,11,19]. The major causes of death in AT include bacterial pneumonia and chronic lung disease [8,20]. A retrospective study analyzed 101 patients who did not have cancer and had chronic respiratory symptoms [21]. This included 36 patients with AT who were alive and 65 patients who died secondary to lung disease. Clinical symptoms included cough, fever, adventitial breath sounds, weight loss, post-tussive emesis, hemoptysis, and pleuritic chest pain. Radiological findings included but were not limited to bronchiectasis, hilar adenopathy, pneumothorax, pleural thickening, and sinusitis. Of the 101 patients, 79 had at least one microbial evaluation of their respiratory secretions, and 61 were positive. Bacterial pathogens isolated included *Pseudomonas aeruginosa*, *Hemophilus influenzae*, *Staphylococcus aureus*, *Streptococcus pneumoniae*, *Escherichia coli*, *Streptococcus viridians*, *Candida albicans*. Of seven patients in whom viral pneumonia was suspected, three had respiratory syncytial virus (RSV) infections, two had clinical and radiological pictures consistent with Varicella pneumonia, and the remaining two had serological evidence of acute EBV infection in association with new pulmonary infiltrates. No opportunistic or fungal pathogens were isolated, with the exception of *Candida albicans*, which was always found in conjunction with other microbes.

A single-center study on 12 patients with AT demonstrated that while patients with low IgG2 had recurrent infections due to *S. pneumoniae*, bacterial pathogens were not demonstrated in the airways of 4 patients with IgG3 deficiency [12]. Interestingly, other studies found no correlation between the frequency of respiratory tract infections and immunoglobulin deficiencies [10,17]. On the other hand, patients with hyper IgM phenotype, where serum IgM levels are normal or elevated and IgG and/or IgA levels are low, presented with a more severe course and shorter survival due to recurrent and severe respiratory infections [13]. 

### 2.2. Bacterial Sepsis and Meningitis in AT

Bacterial sepsis and meningitis are seen as relatively rare in AT. Other factors, such as the presence of malignancy, chemotherapy, as well as indwelling catheters, contribute to the development of invasive infections caused by *Streptococcus pneumoniae*, *Staphylococcus aureus*, and *Pseudomonas aeruginosa* [10]. 

### 2.3. Viral Infections in AT

In one cohort, 44% of patients reported varicella infection, and severe varicella infection requiring hospitalization was reported in 5% of these patients. Warts were reported in 17%, and refractory warts were seen in 7% of total patients [10]. No patient had a cytomegalovirus infection in this cohort. In addition to chronic EBV infection, EBV-associated tumors such as smooth muscle tumors in the liver and nodular sclerosing Hodgkin lymphoma and mucosa-associated lymphoid tissue (MALT Lymphoma) in the parotid gland have been reported in AT [12,22,23,24]. Complications associated with live-viral vaccines for polio, measles, mumps, or varicella zoster have not been reported except for one vaccine-associated poliomyelitis case [25]. However, although causality is unclear, vaccine-strain rubella virus has been isolated in some cutaneous granulomas in patients with primary immunodeficiencies, specifically in DNA repair disorders, including AT [26,27,28]. 

### 2.4. Fungal Infections in AT

Interestingly, despite the high rate of low T cells, infections associated with cellular immunodeficiency are only rarely seen in AT. Moreover, despite the use of frequent antibiotics for respiratory infections, candida infections were reported only occasionally and mostly in the setting of chemotherapy. For example, candida esophagitis was reported in 3 of 100 patients with AT, and in one patient, it was associated with chemotherapy, in another patient, it was concurrent with EBV infection. Additionally, one patient developed candida esophagitis during severe varicella infection [10]. *P. jiroveci* pneumonia was extremely rare in AT [29].

## 3. Nijmegen Breakage Syndrome

Nijmegen breakage syndrome (NBS) is another rare AR defect in DSBR. It is most commonly seen in Slavic populations due to a founder mutation in the gene *NBS-1,* encoding protein named “nibrin” [30]. Nibrin is part of a trimeric complex which also includes MRE11 and RAD50 (the MRN complex). The MRN complex is involved in DSBR by both homologous recombination and nonhomologous end joining. Nibrin recognizes DSB and initiates the relocation of the MRN complex to the sites of DSBs. In addition to a direct role in DNA repair, the MRN complex is also involved in the activation of ATM [31,32]. 

The hallmark of NBS is microcephaly, usually since birth, with normal or mildly impaired psychomotor development. Other cardinal features include a typical facial appearance with a prominent midface, recurrent respiratory infections, chromosomal instability, radiation hypersensitivity, and predisposition to malignancy [30,33]. Some patients may also have café au lait spots, clinodactyly and syndactyly.

Combined humoral and cellular immunodeficiencies are extremely common and highly variable in NBS. In a large cohort, abnormal levels of total serum immunoglobulins were found only in 32 out of 40 patients (80%). The most common immunoglobulin deficiency was combined IgG and IgA deficiency (25%). Interestingly, 36·8% of patients with normal total IgG levels had low IgG subclasses (mainly IgG2 and/or IgG4). Five of 40 patients had markedly elevated concentrations of IgM. Reduced absolute counts of CD4+ T cells were found in 95% and CD8+ T cells in 80% of patients. An elevated number of NK cells was seen in 62.5% of patients. In contrast to AT, in more than 50% of patients with NBS, immunodeficiency may progress over time [34]. Patients with recurrent infections are treated with immunoglobulin replacement therapy and prophylactic antibiotics.

Despite confirmed immunodeficiency, some patients do not develop frequent infections and do not require prophylactic antibiotics or immunoglobulin treatment for many years or until the development of a malignancy.

### 3.1. Bacterial Infections in NBS

The most common infections in NBS include bacterial respiratory tract infections which have been reported in more than 50% of patients [30,33,34,35].

Mycobacterial infections have been reported in only a few patients [35,36,37].

### 3.2. Viral Infections in NBS

Herpes simplex infections with recurrent relapses may be seen in up to 30% of patients with NBS, some of which may be associated with chemotherapy [38].

In a large retrospective study, chronic hepatitis infections were reported in 23% of patients, including 14 children with HBV, three with HCV, two with co-infection with HCV and HBV, and five children with severe and recurrent HZV infection [35].

Other studies also showed that severe or chronic viral infections, especially those caused by lymphotropic and/or hepatotropic viruses such as EBV, CMV, HBV, and HCV, may occur, and they may mimic lymphoma or leukemia [39,40]. In some patients, two and even three viral infections co-existed [35,39]. The median maximum load of EBV DNA was higher in patients with CD3+ T cells of <300 cells/μL compared with those with normal CD3+ T cell levels [39].

In a large prospective study, 38.6% of 57 children with NBS developed lymphatic malignancies [39]. In 68.2% of these patients, viral genetic material was demonstrated before the development of malignancy, including EBV in 63.6%, HBV in 31.8%, HCV in 13.6%, and co-infection with two or three viruses in 8 children. There were statistically significant correlations between monoclonal gammopathy and the persistent presence of EBV DNA and HCV RNA. Although the exact mechanism was not investigated, these findings may suggest that chronic viral (such as EBV or HCV) stimulation may contribute to the development of monoclonal malignancies.

Like in AT, the vaccine-strain rubella virus has been isolated in some cutaneous granulomas in patients with NBS, and hematopoietic stem cell transplantation resulted in scarring resolution of granuloma in two patients [26].

### 3.3. Fungal Infections in NBS

Mucosal candidiasis was reported in as many as 50% of patients with NBS, and some of these patients were on chemotherapy [38,41]. For example, pulmonary fungal infections were suspected in 2.7% of patients and recurrent oral candidiasis in 4.5% [35].

## 4. Bloom Syndrome

Bloom syndrome, a rare AR syndrome, is associated with strong genetic instability characterized by cytogenetic abnormalities such as numerous chromosomal breaks and predisposition to all types of cancers starting at an early age. It is caused by mutations in the *BLM* gene, which encodes a 3′-5′DNA helicase [42]. This is an extremely rare condition of <300 cases registered in the Bloom Syndrome registry. It is more prevalent in populations with high consanguinity rates, such as among Ashkenazi Jews whose ancestors were from Poland or Ukraine. 

Clinical features of Bloom syndrome include prenatal and postnatal growth retardation, microcephaly, and butterfly-shaped facial erythema due to photosensitivity. The most significant manifestation of Bloom syndrome is the development of cancer of any type starting at an early age, including more than one type of independent cancer [43]. 

Patients with Bloom syndrome have variable immunodeficiencies, mostly mild antibody deficiencies. Although they develop recurrent infections of the respiratory and gastrointestinal tracts, they are not susceptible to severe or opportunistic infections [44,45]. Treatment includes immunoglobulin replacement and/or prophylactic antibiotics in patients with recurrent infections. 

## 5. Immunodeficiency, Centromeric Instability and Facial Anomalies (ICF) Syndrome

ICF syndrome is a heterogenous autosomal recessive disorder. The number of genes associated with ICF increased from 1 to 4 in the past couple of years, and they include DNA methyltransferase 3B (*DNMT3B*), zinc-finger and BTB domain-containing 24 (*ZBTB24*), cell division cycle associated 7 (*CDCA7*), and helicase, lymphoid specific (*HELLS*). About 50% of patients with ICF carry biallelic mutations in the *DNMT3B* gene. 

In addition to 3 characteristic findings (variable immunodeficiency, cytogenetic abnormalities, and facial dysmorphism), patients with ICF may present with prenatal and postnasal growth retardation, neurodevelopmental abnormalities, and hematological malignancies [46,47]. Facial features include hypertelorism, flat nasal bridge, epicanthal folds, macroglossia, and micrognathia. 

In one study, hypogammaglobulinemia/agammaglobulinemia was seen in almost all patients regardless of the genotype [47,48]. As a result, recurrent and severe respiratory, gastrointestinal, and skin infections with common organisms are very common in ICF. On the other hand, T cell numbers are normal in the majority of patients during the early years of life, and some patients may develop progressive T cell lymphopenia [47,49]. Opportunistic infections (*Candida albicans*, *Pneumocystis jiroveci*) have been reported in a small number of patients [47,48,50]. ICF rarely presented with severe combined immunodeficiency, and one of those patients died from rubella pneumonia [51,52]. 

## 6. POLE1 Deficiency (FILS Syndrome and IMAGe Syndrome)

Facial dysmorphism, immunodeficiency, livedo, and short stature (FILS) syndrome is a recently described autosomal recessive DNA breakage syndrome caused by a single homozygous intronic variant in *POLE1*, which encodes the catalytic subunit of polymerase E [53,54]. Patients may have intrauterine growth restriction, short limbs, dysmorphic features including malar and mandibular hypoplasia, lacy reticular pigmentation of the face and extremities, recurrent pruritic papular eruptions, small and dysplastic teeth, and feeding aversion [53]. Normal total B, T cells, low class-switched and non-switched memory B cells, and high memory T cells, low NK cells, high IgA, normal total IgG, and low IgM, IgG2, and IgG4 have been reported [53,55]. Recurrent or severe infections were observed in some but not all patients and include chronic rhinosinusitis, purulent otitis media, pulmonary infections, as well as acute CMV infection associated with pancytopenia, splenomegaly, and hepatitis. One patient had recurrent meningitis caused by *Streptococcus pneumonia* [54]. 

Recently a different intronic variant (c.1686+32C > G) in *POLE1* as part of a common haplotype in combination with different loss-of-function variants in trans was described in 15 patients from 12 families [56]. They had clinical features similar to IMAGe syndrome (intrauterine growth restriction, metaphyseal dysplasia, adrenal hypoplasia congenita, and genitourinary anomalies in males), a disorder previously associated with gain-of-function mutations in *CDKN1C*. Five of those patients had increased susceptibility to respiratory infections with lymphopenia and/or low IgM levels. Three patients had low NK cell levels, and one of these patients developed CMV pneumonia and then EBV-associated hemophagocytic lymphohistiocytosis (HLH), requiring allogeneic bone marrow transplantation. Another patient died from an HSV infection at 22 months. 

## 7. POLA 1 Deficiency (X-linked Reticulate Pigmentary Disorder)

X-linked reticulate pigmentary disorder (XLRPD) is a rare disorder characterized by facial dysmorphism, skin manifestations, and immune dysregulation [57,58]. It is caused by a recurrent intronic mutation in the DNA polymerase α gene (*POLA1*). Affected males present with low birth weight, recurrent or persistent diarrhea, recurrent respiratory infections, and feeding difficulties starting before 6 months of age. Sometimes due to recurrent pneumonia and diarrhea, patients may be misdiagnosed as having cystic fibrosis [57,59,60]. All males develop diffuse reticular hyperpigmentation, and females have Baschko’s line hyperpigmentation. Other skin findings that were commonly seen include hypohydrosis and facial telangiectasias. Affected males have coarse facial features, broad upward eyebrows, toe abnormalities, digital clubbing, and dental abnormalities. Infectious manifestations include recurrent bacterial upper and lower respiratory tract infections with common organisms such as *Streptococcus pneumonia*, *Haemophilus influenza*, *Staphylococcus aureus*, *Streptococcus pyogenes*, as well as with *Pseudomonas aeruginosa*, *Mycobacterium avium* and *Candida* spp. leading to chronic lung disease and even death [58,59,60]. The renal abscess has also been reported [60]. 

Upper bacterial respiratory infections are common and usually followed by upper viral respiratory tract infections. Invasive infections such as meningitis, osteomyelitis, sepsis, or skin infections are not common. Recurrent or severe opportunistic infections are not characteristics of XLRPD [58]. 

Many patients develop early-onset gastrointestinal involvement, which is likely due to immune dysregulation rather than infections [58]. 

## 8. MCM4 Deficiency

Mini chromosome maintenance protein 4 (MCM4) is essential in DNA replication and genomic stability. MCM4 AR deficiency has been shown in families with children and adults who had adrenal insufficiency, chromosomal breakage, NK cell deficiency, and growth failure [61,62,63]. Some patients have increased susceptibility to bacterial pneumonia, as well as recurrent infections with herpes simplex virus (HSV) and varicella–zoster virus (VZV) [62,63]. 

## 9. RNF168 Deficiency (Radiosensitivity, Immune Deficiency, Dysmorphic features, Learning Difficulties (RIDDLE) Syndrome)

Ring finger protein 168, also known as RIDDLIN (encoded by *RNF168*), plays a significant role in DSBR [64]. AR deficiencies in RNF168 have been reported in less than 10 patients and are associated with variable immunodeficiency, radiosensitivity, variable neurological abnormalities and dysmorphic features, and some patients present with ATM-like phenotype [65,66]. The immunologic evaluation may include low IgG, IgA, or IgG subclass levels. Chronic and recurrent respiratory infections were reported in many patients, and *Helicobacter pylori* infection was reported in at least one patient. 

## 10. Ligase 1 Deficiency

DNA ligase 1 (LIG1) is the major ligase used in DNA replication. LIG1 deficiency is characterized by growth retardation, variable severity of immune deficiency ranging from early onset hypogammaglobulinemia to severe combined immune deficiency, Omenn-like phenotype, reduced α/βT cells and increased proportions of circulating γδ T cells, and erythrocyte macrocytosis without deficiency of vitamin B12 or folate [67,68,69]. 

Patients may also have photosensitivity, ocular telangiectasias, hepatosplenomegaly, multicystic kidney, cardiac anomaly, and severe transfusion-dependent anemia. Variable immunologic abnormalities include low αβT, B, and NK cells with increased numbers of γδT cells, absent to low IgG, IgM, and IgA levels. Patients develop respiratory viral (Rhinovirus, Adenovirus, Metapneumovirus, and RSV), Herpes zoster infection, gastrointestinal viral (rotavirus), and bacterial urinary tract infections [68,70]. Some of these patients with severe combined immunodeficiency phenotype have been successfully transplanted with hematopoietic stem cell transplantation (HSCT) [69,70]. In contrast to other DNA repair defects, no increased risk of malignancies has been reported in LIG1 deficiency.

## 11. GINS1 Deficiency

This is an AR condition caused by biallelic mutations in GINS complex subunit 1 (*GINS1*) gene and associated with prenatal and postnatal growth failure, dysmorphic face, dermatitis, susceptibility to infections, and autoimmunity. GINS complex plays a critical role in DNA replication [71,72]. Recently, five patients were reported with GINS1 deficiency [72]. Two patients had low T cells, especially low CD8+ T cells. Other patients had low or normal numbers of T and B cells and a normal ratio of naïve and memory CD4+ and CD8+ T cells. In all patients, in vitro T cell proliferation was slightly decreased in response to stimulation with mitogenic phytohemagglutinin (PHA) and low but not abolished in response to recall antigens. They had elevated serum IgA levels, low IgM levels, and low or normal IgG levels. All patients had extremely low numbers of circulating NK cells. Three patients had varicella–zoster virus (VZV), two patients had cytomegalovirus (CMV), and one patient had herpes simplex virus (HSV) infections. One patient had adenovirus and respiratory syncytial virus (RSV) pneumonia, one patient had pneumonia caused by *Aspergillus nidulans* and *Streptococcus agalactiae*. All patients had gastrointestinal infections caused by various microorganisms, including *Enterobacter cloacae*, rotavirus, *Clostridium* spp., *Enterococcus faecalis*, *Escherichia coli*, *Candidca glabrata*, *Acinetobacter lowfii*, and *Pseudomonas aeruginosa*.

## 12. NSMCE3 Deficiency

The NSE3 Homolog, SMC5-SMC6 Complex Component (*NSMCE3*) gene encodes a component of the SMC5/SMC6 complex, which is essential for DNA damage response and chromosome segregation during cell division. NSMCE3 deficiency, also known as lung disease, immunodeficiency, and chromosome breakage syndrome (LICS), is an AR disorder characterized by failure to thrive, immune deficiency, and progressive pediatric lung disease due to viral infection that may be fatal [73]. Other features may include severe insulin resistance, eczema, axial hypotonia, and mild psychomotor retardation. According to a recent study of 4 children with NSMCE3 deficiency, patients had elevated IgM, normal or elevated IgA and IgG, and low T cells, specific antibody deficiency, as well as decreased lymphocyte proliferation after mitogen and antigen stimulation [73]. Reported infections in these patients included viral and bacterial pneumonia and osteomyelitis, listed in Table 1.

## 13. MCM10 Deficiency

Minichromosomal maintenance complex member 10 encoded by *MCM10* is a highly conserved component of the replisome that binds directly to the MCM2-7 complex, CDC45, and then DNA and plays an important role in NK cell development. A compound heterozygous mutation in *MCM10* was demonstrated in a single patient who presented at 16 months of age with fever, organomegaly, diarrhea, and CMV infection, which was eventually fatal [74]. The patient’s laboratory evaluation showed slightly low T and B cells but profoundly low NK cells. 

## 14. POLE2 Deficiency

AR POLE2 deficiency was recently described in a 5-year-old Saudi-origin boy who had a history of omphalitis and erythroderma in the neonatal period, systemic Bacillus Calmette-Guerin (BCG) infection after immunization, multiple respiratory infections, diabetes mellitus at age 5 months of age, severe dyspnea and hypoxia, hepatomegaly, and hypothyroidism at 8 months of age [75]. He also had dysmorphic features, including a low anterior hairline, flat supraorbital ridges, downturned corners of the mouth, and a short philtrum. The immunologic evaluation revealed agammaglobulinemia with an absence of circulating B cells, T-cell lymphopenia, low NK cells, and neutropenia. He died following treatment-resistant HLH. 

## 15. Prevention of Infections

Like in other inborn errors of immunity, in patients with DNA repair defects, prevention of infections is tailored based on the clinical presentation and type and level of the immune deficiency. For example, in a large cohort of children with AT, at least 50% of 61 patients with no ATM activity developed low IgG and recurrent infections. Ten patients were placed on immunoglobulin replacement therapy (IgRT), and 30 received prophylactic antibiotics [76]. On the other hand, only 4 of 19 patients with some residual ATM activity developed recurrent infections. Among this group, one patient had low IgG2, and six patients had specific antibody deficiency. None of these patients were placed on IgRT; only one received prophylactic antibiotic treatment.

According to a systemic review that included 18246 patients with AT, IgRT was administered in 819 (<5%) patients, and 332 patients (<2%) received prophylactic antibiotics [16]. Another study of 100 patients with AT reported that 10% of patients received IVIG [10]. In this study, three patients received, in addition to IVIG, *P*. *jerovici* prophylaxis with trimethoprim-sulfamethoxazole because of low CD4-lymphocyte counts.

In a study of 149 children with NBS where low IgG and recurrent respiratory infections were observed in 62%, and at least 50%, respectively, and 68% of children received IVIG [35]. Antibiotic prophylaxis was given in 5 patients with chronic bronchitis. In this cohort, prophylaxis against viral, fungal, and *P*. *jiroveci* infections was not routinely recommended or implemented.

IgRT, as well as prophylactic antimicrobials, have also been reported in other DNA repair defects, such as ICF, ligase 1 deficiency, RNF168 deficiency, GINS1 deficiency, and POLE2 deficiency [47,51,65,70,72,75,77,78].

In addition to systemic antibiotic treatments, inhaled antibiotic therapy has been reported in a patient with POLA1 deficiency who continued to develop recurrent pneumonia due to permanent lung damage [58].

In patients with combined immunodeficiency or malignancy, HSCT can be a treatment of choice. For example, in one large cohort, 11 of 149 patients with NBS underwent HSCT due to resistant, relapsing, or secondary malignancies, and three children received HSCT mainly because of severe immunodeficiency [35]. Of these 14 patients, nine (71%) remained alive with good immune reconstitution after a median follow-up of 6 years (range, 1.4–11 years), but five patients died because of relapsing of malignancy or infections. HSCT has also been reported in patients with ICF and ligase 1 deficiency [47,51,70,77,78,79]. One patient with MCM10 deficiency received bone marrow transplantation because of HLH but died from severe CMV infection [74].

Because of severe permanent lung damage, one patient with NSMCE3 deficiency received cadaveric lung transplantation at 15 months of age [73].

## 16. Discussion

Inherited defects in DNA repair pathways may lead to rare disorders such as ataxia telangiectasia, Nijmegen breakage syndrome, Bloom syndrome, and even more rare disorders reported in only one or less than 10 patients. In addition to immunodeficiencies, clinical manifestations include intra-uterine and/or extra-uterine growth delay, microcephaly, facial dysmorphism, and developmental delay in many patients. Some patients have skin abnormalities such as telangiectasia, butterfly rash, eczema, or hyperpigmentation. Another important feature of most of these conditions is the increased risk of malignancies which may also be associated with viral infections. Since both the humoral and cellular immune systems are affected, the most frequent infections seen in these patients are common infections seen in combined immunodeficiencies, and they include recurrent or severe respiratory tract infections. In some disorders severity of infections does not correlate with the severity of immunodeficiency. For example, in ataxia telangiectasia, neurologic abnormalities contribute to the development of recurrent pneumonia seen in patients with normal or almost normal immunoglobulin levels. In some defects, such as deficiencies of MCM4, GINS1, and MCM10, where there is a significant susceptibility to recurrent and severe viral infections, the pathogenesis may be explained by severely defective NK cells.

It should be noted that some of these conditions, such as deficiencies of RNF168, ligase 1, GINS1, and NSMC3, were reported in only <10 patients, and only a single patient was described on each PMS2, MCM10, POLE2, and Hebo deficiencies. Moreover, in some of these conditions, there have been non-overlapping phenotypes in individuals with similar genotypes. Therefore, it is essential to identify additional patients to understand consistent clinical phenotypes and better define the clinical spectrum of these rare disorders.

In summary, patients with DNA repair defects require significant medical attention for a variety of symptoms, including susceptibility to recurrent and severe infections. Since in most patients, increased risk of malignancy and its complications, such as susceptibility to toxicities to DNA-damaging therapies, are major concerns, diagnosing DNA repair defects early is critical. Therefore, recurrent infections in children who also have other findings listed above should alert physicians to investigate these patients for DNA repair defects. 

Recurrent infections may be prevented by IgRT, prophylactic antimicrobials, and HSCT in some patients. 

**Table 1:** DNA repair defects (excluding defects associated with severe combined immunodeficiencies and Fanconi anemia) and reported infectious complications.

Disease	Gene	Inheritance	Reported Infections	Other Findings
**Ataxia-telangiectasia**	*ATM*	AR	Upper and lower respiratory tract infections *P. aeruginosa*, *H. influenzae*, *S. aureus*, *S. pneumoniae*, *E. coli*, *S. viridians*, *C. albicans,* RSV, Varicella, EBV Invasive infections with *S. pneumoniae*, *S. aureus*, and *P. aeruginosa* VZV infections, warts Vaccine-associated polio (1 case) Vaccine-strain rubella virus isolated in lesions of granulomatous dermatitis HHV6, *H*. *parainfluenza* EBV-associated malignancies	Ataxia, telangiectasia, elevated alpha-feto protein, increased risk of lymphoid and other malignancies, increased radiosensitivity, and chromosomal instability
**Nijmegen breakage syndrome**	*NBS1*	AR	Bacterial respiratory tract infections Mycobacterial infections (only a few patients) Recurrent HSV Severe or chronic EBV, CMV, HBV, HCV Recurrent VZV Vaccine-strain rubella virus isolated in lesions of granulomatous dermatitis Mucosal candidiasis	Progressive microcephaly, dysmorphic facies with prominent midface; lymphomas and solid tumors; increased radiation sensitivity; chromosomal instability
**Bloom syndrome**	*BLM*	AR	Respiratory and gastrointestinal infections with common microorganisms. No increased susceptibility to opportunistic or severe infections.	Short stature, dysmorphic facies sun-sensitive erythema; marrow failure; leukemia, lymphoma; chromosomal instability.
**Immunodeficiency with centromeric instability and facial anomalies** **(ICF types 1, 2, 3, 4)**	*DNMT3B*	AR	Recurrent or severe respiratory, gastrointestinal, and skin infections with common organisms Opportunistic infections (*C. albicans*, *P*. *Jiroveci*) rare Rubella pneumonia (1 patient)	Facial dysmorphic features, developmental delay, macroglossia; malabsorption; cytopenias; malignancies
	*ZBTB24*	AR		
	*CDCA7*	AR		
	*HELLS*	AR		
**POLE1 (Polymerase ε subunit 1) deficiency (FILS syndrome and IMAGe syndrome)**	*POLE1*	AR	Recurrent respiratory tract infections Viral infections (CMV, HSV, EBV) Recurrent meningitis caused by *S. pneumonia* (1 patient)	Facial dysmorphism, livedo, short limbs, dysmorphic features
**X-linked reticulate pigmentary disorder (POLA1 deficiency)**	*POLA1*	XL	Recurrent bacterial upper and lower respiratory tract infections with *S*. *pneumonia, H. influenza, S. aureus, S. pyogenes, P. aeruginosa*, *M*. *avium* and *Candida* spp Renal abscess (1 patient)	Hyperpigmentation, characteristic facies, hypohydrosis, facial telangiectasia
**MCM4 deficiency**	*MCM4*	AR	Bacterial pneumonia Viral infections (HSV, VZV)	NK cells: low number and function, short stature; B cell lymphoma; adrenal insufficiency
**RNF168 deficiency (Radiosensitivity, Immune Deficiency, Dysmorphic features, Learning difficulties -RIDDLE syndrome)**	*RNF168*	AR	Chronic and recurrent respiratory tract infections *H*. *pylori* infection (1 patient)	Short stature, mild defect of motor control to ataxia; normal intelligence to learning difficulties; mild facial dysmorphism to microcephaly; increased radiosensitivity
**Ligase I deficiency**	*LIG1*	AR	Recurrent bacterial and viral infections	Failure to thrive, photosensitivity, erythrocyte macrocytosis No increased risk of malignancy
**GINS1 deficiency** **(5 patients)**	*GINS1*	AR	Viral infections (VZV, CMV, HSV) All had gastrointestinal infections (*E*. *cloacae*, rotavirus, *Clostridium* spp., E. *faecalis*, *E. coli*, *C. glabrata*, *A. lowfii*, *P. aeruginosa* 1 patient with pneumonia caused by *A*. *nidulans*, and *S*. *agalactiae*	Intra and extra-uterine growth failure, dysmorphic face, dermatitis, autoimmunity, NK cells very low
**NSMCE3 deficiency**	*NSMCE3*	AR	Pneumonia with common virus and bacteria Osteomyelitis with *C*. *albicans* Central line infection with *Serratia marcences*, *E*. *coli*, *E*. *fecalis*	Failure to thrive, thymic hypoplasia; chromosomal breakage, axial hypotonia, eczema
**PMS2 Deficiency**	*PMS2*	AR	Recurrent infections (1 patient), not characterized	Café-au-lait spots; lymphoma, colorectal carcinoma, brain tumors
**MCM10 deficiency** **(1 patient)**	*MCM10*	AR	Fatal CMV infection	Very low NK cells, HLH-like presentation
**POLE2 (Polymerase ε subunit 2) deficiency** **(1 patient)**	*POLE2*	AR	Respiratory infections, Systemic BCG infection	Autoimmunity (type 1 diabetes), hypothyroidism, facial dysmorphism
**ERCC6L2 (Hebo deficiency)** **(6 patients)**	*ERCC6L2*	AR	None reported	Facial dysmorphism, microcephaly; bone marrow failure

## Data Availability

Not applicable.
